# A Flexible Syntaxin Solves the Mystery of the SNAREd Munc

**DOI:** 10.1371/journal.pbio.0040359

**Published:** 2006-09-26

**Authors:** Richard Robinson

Fusion of two plasma membranes is central to exocytosis, the process by which a cell secretes neurotransmitters, digestive enzymes, and other products. If you believe the simple diagrams in introductory biology textbooks, you’d think this fusion occurs as soon as two membranes touch. Not so—in fact, membrane fusion requires interaction among a complex set of proteins in the membranes, collectively termed SNARE proteins. SNAREs are assisted by a second group, called SM proteins, which bind to them and help promote their functions.

Among the SM proteins, one, called Munc18-1, has stood out as something of an oddball. When the others bind to their respective SNAREs, they leave the SNAREs in an open conformation, available for interacting with others and forming the complexes that drive membrane fusion. In contrast, Munc18-1 appears to fold its SNARE, syntaxin 1, into a closed conformation, making it unavailable for binding to other SNAREs. But this result has been obtained only in membrane-free solutions, and the behavior of membrane-bound Munc18-1 has been a mystery. A new study by Felipe Zilly, Thorsten Lang, and colleagues resolves the mystery of the syntaxin–Munc18-1 interaction and explains how their binding promotes interactions with other SNAREs.

The authors performed their experiments in sheets of membrane, prepared by disrupting cells, which mimic the native biochemical environment of Munc18-1 much better than membrane-free solutions. First they showed that syntaxin must be able to close to bind Munc18-1; when mutated to prevent closure, syntaxin bound virtually no Munc18-1. But proteins are flexible molecules, and it is possible that syntaxin needn’t stay closed while it is bound to Munc18-1, and that adopting a more open conformation while bound would promote its linkage to other SNAREs.

**Figure:**
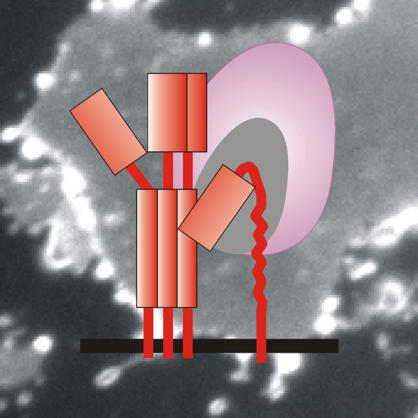
Munc18-1 (purple) binds to a half-open syntaxin (red), which is primed to form SNARE complexes with synaptobrevin and SNAP-25 in plasma membrane sheets (seen in the background image).

To test this possibility, the authors added another SNARE, synaptobrevin, to the mix. Synaptobrevin is a known partner for syntaxin, and it has been shown that the addition of synaptobrevin drives syntaxin (without Munc18-1) in conjunction with SNAP-25 (the third SNARE essential for neuroexocytosis) into SNARE complexes. They reasoned that adding synaptobrevin to syntaxin–Munc18-1 would not drive syntaxin into SNARE complexes if syntaxin remained closed. Conversely, if syntaxin could partially open while bound to Munc18-1, it would be able to interact with synaptobrevin and join the SNARE complex. And this is what they found—when synaptobrevin was added, syntaxin unhitched from Munc18-1 and joined the SNARE complex, involving most likely also SNAP-25. Finally, by deleting SNAP-25, the authors verified its essential role in displacing Munc18-1 from syntaxin, suggesting there is an intermediate complex formed by Munc18-1, syntaxin, synaptobrevin, and SNAP-25.

These results not only shed light on the actual function of Munc18-1, but allow the development of a more coherent picture of SM proteins, in which Munc18-1 is no longer the oddball. They also illustrate the complex interactions among proteins that bring about such a “simple” process as membrane fusion.

